# Comparative analysis of treatment outcomes and risk factors associated with different combination antimicrobial regimens in patients with multidrug-resistant Acinetobacter baumannii pneumonia

**DOI:** 10.3389/fcimb.2026.1819679

**Published:** 2026-06-19

**Authors:** Meng Xu, Mingxing Liu, Xuebin Tang, Xiaoying Wu

**Affiliations:** 1 Clinical Pharmacology, Affiliated Hospital of Qinghai University, Xining, Qinghai, China; 2 Department of Emergency and Critical Care, Jiading Branch of Shanghai General Hospital, Shanghai Jiaotong University School of Medicine, Shanghai, China; 3 Department of Respiratory and Critical Care Medicine, Tongren People’s Hospital, Tongren, Guizhou, China; 4 Department of Emergency Medicine, Shanghai Fourth People’s Hospital, School of Medicine, Tongji University, Shanghai, China

**Keywords:** antibiotic resistance, hospital-acquired pneumonia, mortality rate, multidrug-resistant *Acinetobacter baumannii*, risk factors

## Abstract

**Background:**

Multidrug-resistant *Acinetobacter baumannii* (MDRAB) pneumonia is a major cause of mortality in hospital-acquired infections due to extensive drug resistance and limited therapeutic options. Optimizing treatment and identifying mortality-related factors are urgently needed.

**Objective:**

To compare the efficacy of different antibacterial regimens in MDRAB pneumonia and determine independent risk factors for 28-day all-cause mortality.

**Methods:**

This single-center retrospective cohort study included 142 MDRAB pneumonia patients from February 2022 to February 2025. Patients were divided into three groups: Group A, sulbactam-containing combinations (n = 63); Group B, polymyxins + tigecycline (n = 45); Group C, other regimens (n = 34). Outcomes included bacterial resistance patterns, 28-day mortality, dynamic PCT and CRP levels, APACHE II and SOFA scores, and lymphocyte subsets. Multivariate logistic regression identified mortality predictors.

**Results:**

Group A showed the lowest resistance to cefoperazone/sulbactam (30.16%) and the highest sensitivity (71.43%, both P< 0.05). Group B had the highest tigecycline resistance (31.11%). The 28-day mortality rates were 39.68% (A), 60.00% (B), and 64.71% (C) (P = 0.028). Independent mortality predictors were APACHE II, SOFA, and CD4+/CD8+ ratio (all P< 0.01). On day 7, Group A had the lowest PCT, while Group C showed the smallest declines in PCT and CRP. Group A exhibited the highest CD4+/CD8+ ratio.

**Conclusion:**

Sulbactam-based combination therapy was associated with improved pathogen clearance and survival in this retrospective cohort. Mortality is closely linked to initial disease severity and early organ dysfunction. Dynamic changes in PCT and CD4+/CD8+ ratio have significant prognostic value.

## 1 Introduction

In the fields of critical care medicine and respiratory system infections, pneumonia caused by multidrug-resistant Acinetobacter baumannii (MDRAB), especially hospital-acquired pneumonia (HAP) and ventilator-associated pneumonia (VAP), has already become a pressing and increasingly severe global health threat (Montrucchio et al., 2024). Acinetobacter baumannii exhibits remarkable colonization and transmission potential in hospital environments, particularly in intensive care units (ICUs), due to its strong environmental survival capabilities, the tendency to form biofilms on medical devices, and the efficient mechanism for acquiring and disseminating drug-resistant genes (Duan et al., 2024). What is particularly worrying is the rapid development of its drug resistance. MDRAB (multi-drug resistant Acinetobacter baumannii) strains, which show resistance to almost all commonly used broad-spectrum antibacterial drugs, including carbapenems, have spread widely worldwide (Ibrahim et al., 2021). Numerous monitoring reports indicate that the detection rate of carbapenem-resistant Acinetobacter baumannii (CRAB) has exceeded 70% in many regions, and even pan-drug-resistant strains (XDRAB) that are resistant to almost all available drugs have emerged (Jalali et al., 2023; Liu et al., 2024). This severe situation of drug resistance not only severely limits the clinical treatment options, significantly prolongs the hospitalization period of patients, and brings about a heavy medical economic burden, but also directly endangers patients’ lives, posing a huge challenge to the prevention of nosocomial infections and anti-infection treatment.

The clinical severity of MDRAB pneumonia is primarily reflected in its extremely high fatality rate (Zhou et al., 2019; Alenazi et al., 2023). A large number of clinical studies have consistently confirmed that, compared with pneumonia caused by sensitive bacterial strains or other common pathogens, the overall mortality rate and attributable mortality rate of patients with MDRAB pneumonia have significantly increased. This risk is particularly prominent in patients with severe underlying diseases, immunosuppression, or in critical conditions (Xu et al., 2023). The high mortality rate is the result of a complex interplay of various factors: the pathogens themselves have considerable invasiveness and pathogenicity; drug resistance leads to a significant delay or even absence of effective initial antibacterial treatment; the patient population often has complex comorbidities and an immunosuppressive background; and the infection itself can trigger an uncontrollable systemic inflammatory response storm (Alrahmany et al., 2022; Frattari et al., 2024; Černiauskienė and Vitkauskienė, 2025). It is worth noting that MDRAB infections are often not isolated incidents. They are typically the hallmark complications experienced by patients after undergoing major trauma, complex surgeries, prolonged hospitalization, receiving broad-spectrum antibiotic treatments, and undergoing invasive mechanical ventilation, among other high-risk medical interventions. These complex background factors themselves already indicate an unfavorable prognosis, making it extremely difficult yet crucial to precisely isolate the independent contribution of the infection to mortality and to identify the modifiable risk factors within it.

When dealing with MDRAB pneumonia, clinicians often find themselves in a situation where the treatment options are extremely limited and challenging. The currently available drugs mainly include polymyxins (such as polymyxin B and colistin), tigecycline, sulbactam and its combination preparations (such as ampicillin/sulbactam and cefoperazone/sulbactam), as well as some still active aminoglycoside drugs (Karakonstantis et al., 2020). However, all these drugs have significant flaws or uncertainties. Polymyxins have clear risks of nephrotoxicity and neurotoxicity, and their ability to penetrate lung tissue and their efficacy in killing bacteria within biofilms are often questioned (Ayoub Moubareck and Hammoudi Halat, 2020). When tigecycline is used to treat pneumonia, there is a concern that the blood drug concentration may be too low, resulting in poor clinical efficacy (Park et al., 2024). The resistance to sulbactam has increased, and the efficacy of monotherapy is often unsatisfactory (Wenzler et al., 2017). Aminoglycoside antibiotics are limited in their widespread application due to their significant nephrotoxicity and ototoxicity (Le et al., 2023). Due to the aforementioned treatment challenges, the combined medication strategies based on the results of *in vitro* drug susceptibility synergy tests and limited clinical observations (such as colistin combined with sulbactam, colistin combined with tigecycline, colistin combined with carbapenems, etc.) have become common approaches in clinical practice aimed at improving efficacy, overcoming drug resistance, and possibly reducing the toxicity of single drugs. However, there is still a lack of high-level evidence from large-scale, well-designed prospective randomized controlled trials (RCTs) to support key questions such as which combination actually has the optimal efficacy and safety, the exact clinical value of aerosol inhalation, and how the optimal dosage and treatment course should be determined. The current recommendations in domestic and international guidelines are mostly based on low-quality evidence or expert consensus, and clinical decisions largely rely on individual doctors’ experience judgments and interpretation of *in vitro* drug susceptibility results, resulting in considerable subjectivity and uncertainty. This ambiguity in treatment strategies directly hinders the effective improvement of patient prognosis.

Although the high mortality risk of MDRAB pneumonia is widely recognized, accurately identifying the independent risk factors that affect patient mortality, and objectively evaluating the exact impact of different treatment strategies on survival outcomes, remain important gaps and points of contention in current research (Zhou et al., 2019). Previous studies have often been limited by small sample sizes, short observation periods, or insufficiently adjusting for numerous potential confounding factors in multivariate analyses, such as the severity of underlying diseases in patients (quantified by APACHE II or SOFA scores), the level of organ support (especially mechanical ventilation), and the presence of co-infections with other pathogens, etc. Regarding the relative superiority or inferiority of specific antibiotic combinations (such as polymyxin + teicoplanin vs. polymyxin + sulbactam vs. regimens based on sulbactam-containing compound preparations), the results of different studies often contradict each other (Qu et al., 2020; Zha et al., 2023). Apart from the anti-infection treatment plan itself, in-depth exploration of which patient characteristics (such as advanced age, specific comorbidities, high disease severity score), infection-related factors (such as whether there is sepsis, the severity and type of pneumonia), laboratory indicators (such as inflammatory markers C-reactive protein, procalcitonin levels and their dynamic changes), or medical intervention factors (such as duration of mechanical ventilation) can independently predict the risk of death holds more direct clinical practice significance. Systematically analyzing these risk factors not only helps in early identification of high-risk patients to implement more proactive monitoring and intervention measures, but also provides crucial scientific basis for optimizing clinical decision-making paths and rationally allocating limited medical resources.

In order to deeply explore the aforementioned core issues, fill the gaps in existing knowledge, and provide more targeted local evidence for clinical practice, we conducted this retrospective cohort study. We systematically collected and analyzed the complete clinical medical records, microbiological data, detailed treatment records, and final prognosis information of 142 adult patients with MDRAB pneumonia who were diagnosed by microbiology in our hospital from February 2022 to February 2025 over a three-year period.

This study aims to rigorously analyze and compare different antibacterial drug treatment strategies (including single drug therapy and various combination therapy schemes) with the final clinical outcomes of patients. By constructing a multivariate regression model, it systematically screens and confirms the independent predictive factors significantly related to the risk of patient death, and attempts to establish a risk prediction model. It also assesses the therapeutic benefits and potential safety issues of aerosolized antibacterial drugs in clinical applications.

## 2 Research subjects and methods

### 2.1 Research subjects

**Figure 1:**
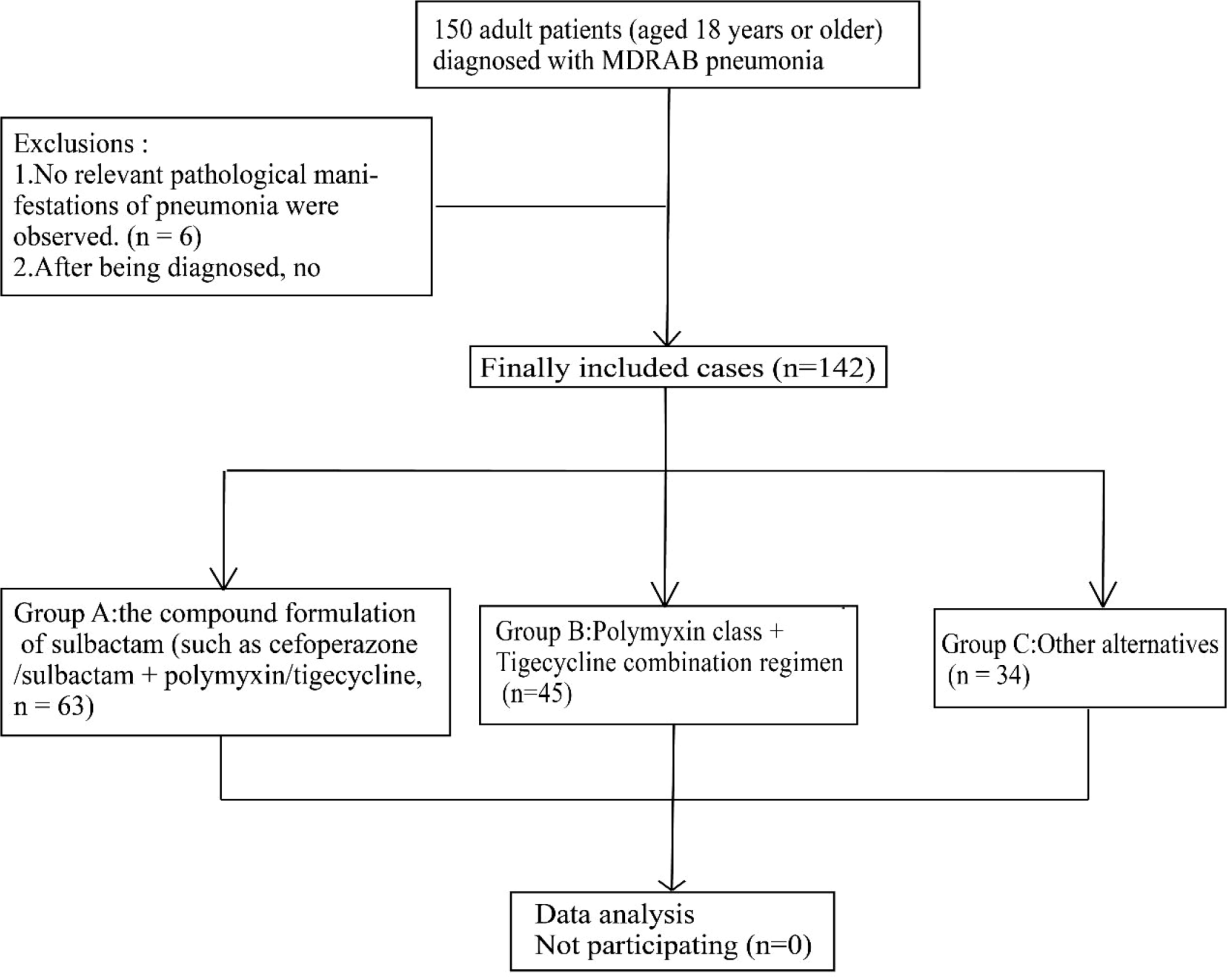
Flowchart.

This study adopted a single-center retrospective cohort study design. The study subjects were adult patients (aged ≥ 18 years) who were hospitalized in our hospital and were diagnosed with MDRAB pneumonia through microbiological examination from February 1, 2022 to February 28, 2025. MDRAB was defined as a strain of Acinetobacter baumannii that was resistant to three or more structurally distinct antibacterial drugs, including carbapenems (imipenem or meropenem). The diagnosis of pneumonia required both clinical criteria (new or progressive pulmonary infiltrates accompanied by at least two of the following manifestations: fever > 38 °C, purulent respiratory secretions, leukocytosis or leukopenia, or deterioration of oxygenation) and microbiological criteria (qualified lower respiratory tract specimens, such as bronchoalveolar lavage fluid, protective brush, or deep sputum culture, isolated MDRAB, and reaching a clinically significant threshold concentration). A total of 142 patients meeting the criteria were ultimately included in the study. See **Figure 1**.

### 2.2 Inclusion and exclusion criteria

Inclusion criteria (Lan et al., 2024)**:** (1) Age ≥ 18 years; (2) Meets the clinical and microbiological diagnostic criteria for MDRAB pneumonia as described above; (3) Was diagnosed and received targeted anti-infective treatment during hospitalization; (4) Has complete clinical medical records, microbiological reports, treatment plan records, and key laboratory test results.

Exclusion criteria (Alrahmany et al., 2022): (1) Only colonization of Acinetobacter baumannii (without corresponding clinical manifestations of pneumonia); (2) Complicated with other uncontrollable fatal non-infectious diseases (such as terminal malignant tumors, severe irreversible neurological diseases) with an expected lifespan of less than 72 hours; (3) After being diagnosed with MDRAB pneumonia, no targeted antibacterial treatment was received or the treatment duration was less than 48 hours and the patient was automatically discharged or gave up treatment; (4) Key clinical data (such as underlying diseases, details of treatment plans, 28-day survival status) are severely lacking and cannot be evaluated.

### 2.3 Research methodology

The complete data of patients meeting the inclusion criteria were retrospectively collected through the hospital electronic medical record system (EMR) and laboratory information system (LIS). The data collection was carried out by researchers who had received unified training, using a pre-designed standardized data collection form to ensure the consistency and accuracy of information extraction. The collected contents included:

1. Baseline information: demographic data (age, gender), underlying diseases and comorbidities (such as chronic obstructive pulmonary disease, diabetes, chronic kidney disease, malignant tumors, immunosuppressive state, etc.), reasons for admission, and ICU admission status.
2. Infection-related information: type of pneumonia (hospital-acquired pneumonia HAP, ventilator-associated pneumonia VAP), diagnosis time, infection site (whether combined with bacteremia), length of hospital stay before infection onset, mechanical ventilation status and duration.
3. Microbiological data: Source of strain isolation, identification results, analysis results of the strain’s resistance spectrum (main detection indicator 1). Treatment information: Detailed record of the treatment plan, including: specific types of antibacterial drugs used (polymyxin class, tigecycline, sulbactam-containing compound preparations, aminoglycosides, carbapenems, etc.), treatment mode (monotherapy vs. combination therapy; specific combination schemes, such as polymyxin + tigecycline, polymyxin + sulbactam compound preparations, tigecycline + sulbactam compound preparations, etc.), the start time of anti-infection treatment (relative to the time when the pneumonia diagnosis criteria were met), total treatment course, and other supportive treatments (such as infection site clearance operations, immunomodulatory treatments, etc.).
4. Severity of the disease and organ function indicators: APACHE II score (secondary detection indicator 5): Calculate the worst value within 24 hours of pneumonia diagnosis. SOFA score (secondary detection indicator 5): Calculate the worst value within 24 hours of pneumonia diagnosis.
5. Laboratory indicators: Dynamic levels of serum procalcitonin (PCT) and C-reactive protein (CRP) (secondary detection indicators 4): Record the values at the time of diagnosis (baseline), on the 3rd day of treatment, and on the 7th day (if no test on that day, take the closest value before or after, but within ±1 day). The detection method is electrochemiluminescence. Absolute values of lymphocyte subsets and CD4^+^/CD8^+^ ratio (secondary detection indicators 6): Record the absolute counts of peripheral blood lymphocyte subsets (total T lymphocytes CD3+, helper T cells CD3^+^CD4^+^, cytotoxic T cells CD3^+^CD8^+^, B cells CD19^+^, NK cells CD3^-^CD16^+^CD56^+^) and the CD4^+^/CD8^+^ ratio at the time of diagnosis (baseline). The detection method is flow cytometry.
6. Outcome indicators:

Primary outcome: 28-day all-cause mortality rate (primary outcome measure 2): Defined as death due to any cause within 28 days from the date of MDRAB pneumonia diagnosis.

### 2.4 Detection indicators

#### 2.4.1 Main detection indicators

1. Analysis of the drug resistance spectrum of the strains: conducted using the VITEK 2 system.
2. 28-day all-cause mortality rate: death due to any cause within 28 days from the date of diagnosis.

#### 2.4.2 Secondary detection indicators

1. Dynamic levels of serum inflammatory markers: The values of PCT and CRP at baseline, on the 3rd day of treatment, and on the 7th day (detected by electrochemiluminescence method).
2. Severity of the disease and organ function: APACHE II score and SOFA score (the highest value within 24 hours after pneumonia diagnosis).
3. Assessment of immune function: Absolute counts of baseline lymphocyte subsets (CD3^+^, CD3^+^CD4^+^, CD3^+^CD8^+^, CD19^+^, CD3^-^CD16^+^CD56^+^) and the ratio of CD4^+^/CD8^+^ (detected by flow cytometry).

### 2.5 Sample size consideration

This was a retrospective observational study with three treatment groups. Given that the primary comparison of interest was between the sulbactam-based regimen (Group A) and the other two regimens (Groups B, C), we initially performed a sample size calculation based on a two-arm comparison using parameters from a previous study by Ungthammakhun et al (Ungthammakhun et al., 2024), (HR = 0.54,*P* = 0.0110). Using the formula for two independent samples (Hedges and Olkin, 1985), with α = 0.05 (two-sided), β = 0.2 (power 80%), the minimum required sample size was 136 patients. To account for potential dropouts or missing data, we enrolled 142 eligible patients.

However, we acknowledge that a formal sample size calculation for a three-arm, non-randomized, unbalanced cohort is methodologically complex and was not performed *a priori*. Instead, the achieved sample size of 142 — with 63 in Group A, 45 in Group B, and 34 in Group C — provides sufficient events (75 deaths) to support multivariable regression analyses (recommended minimum of 10 events per variable) [reference], as well as propensity score matching and subgroup analyses. Therefore, the study should be considered exploratory, and findings are presented with appropriate adjustments for baseline imbalances.

### 2.6 Statistical methods

All data were statistically analyzed using SPSS 26.0 (IBM Corp., Armonk, NY, USA) and R version 4.2.0 (R Foundation for Statistical Computing, Vienna, Austria). For descriptive analysis, continuous variables were expressed as mean ± standard deviation (SD) or median with interquartile range (IQR) based on normality, and categorical variables as frequency (percentage). Differences among the three groups were compared using one-way ANOVA (or Kruskal-Wallis test for non-normal data) for continuous variables and chi-square test for categorical variables.

To address baseline imbalances among treatment groups, we performed propensity score matching (PSM). Propensity scores were calculated using a logistic regression model that included treatment group (Group A vs. Group B; and Group A vs. Group C separately) as the dependent variable and the following baseline covariates as independent variables: age, APACHE II score, SOFA score, pneumonia type (VAP/HAP), ICU admission, mechanical ventilation at diagnosis, bacteremia, and pre-infection hospital days. One-to-one nearest-neighbor matching was performed with a caliper width of 0.2 SD of the logit of the propensity score. Standardized mean differences (SMD) were used to assess balance; an SMD< 0.1 was considered well-balanced. After matching, 28−day mortality rates were compared between matched cohorts using McNemar’s test, and survival curves were estimated using Kaplan−Meier method with log−rank test.

Multivariable logistic regression analysis was performed on the original cohort to identify independent risk factors for 28-day all-cause mortality. Covariates with p< 0.10 in univariate analysis or those considered clinically important (including treatment group, APACHE II, SOFA, CD4^+^/CD8^+^ ratio, and bacteremia) were entered into a forward stepwise logistic regression model. Results are presented as adjusted odds ratio (OR) with 95% confidence interval (CI). For continuous variables, the ORs were calculated per unit increase. Because the CD4^+^/CD8^+^ ratio ranges from 0.2 to 2.5, its OR per 1.0 unit increase would be extremely small. To present clinically interpretable results, we re-scaled this variable by multiplying by 10, so that the OR corresponds to a 0.1-unit increase in the CD4^+^/CD8^+^ ratio. A two-sided p< 0.05 was considered statistically significant for all analyses.

## 3 Result

### 3.1 Baseline information

This study initially included 142 adult patients (aged ≥ 18 years) who were hospitalized in our hospital and were diagnosed with MDRAB pneumonia through microbiological examination from February 1, 2022 to February 28, 2025. These patients were divided into three groups for a retrospective study. There were no significant differences in age (mean 55.27 years vs 56.71 years vs 57.15 years) and gender ratio (male 61.9% vs 57.78% vs 64.71%) among the three groups (*P* > 0.05), indicating that the treatment groups were not influenced by demographic factors. There was no preference for treatment groups in terms of smoking status (*P* = 0.891), alcohol history (41.27% vs 40.00% vs 41.18% in groups A, B, and C respectively), and no statistical difference (*P* > 0.05). The distribution of underlying diseases (chronic lung disease, diabetes, chronic kidney disease, and malignant tumors) was balanced among the three groups (*P* > 0.05). Regarding the type and severity of pneumonia: the proportion of ventilator-associated pneumonia (VAP) in group B was significantly higher than that in groups A and C (55.56% vs 34.92%/29.41%, *P* = 0.033), suggesting that the infection in this group was more associated with mechanical ventilation. The length of hospital stay before infection in group B was significantly longer than that in group A (16.47 days vs 12.10 days, *P* < 0.001), reflecting a longer medical exposure history. In terms of ICU and mechanical ventilation, the proportion of admission to ICU in group B was significantly higher than that in group A (95.56% vs 82.54%, *P* = 0.044). The rate of mechanical ventilation at diagnosis was significantly higher in group B than in groups A and C (95.56% vs 76.19%/76.47%, *P* = 0.020). There were significant differences in disease severity and organ function critical scores: APACHE II score: the score of group B (25.24 ± 6.14) was significantly higher than that of group A (19.38 ± 5.62) and group C (19.96 ± 7.27) (*P* < 0.001), indicating that the overall disease severity of group B was the highest. SOFA score: the median score of group B (10.17 points) was significantly higher than that of group A (8.05 points) and group C (8.32 points) (*P* < 0.001), reflecting more severe organ dysfunction. See **Table 1**.

**Table 1:** The demographic and clinical characteristics of the three groups of patients.

baseline		Group A (n = 63)	Group B (n = 45)	Group C (n = 35)	Test	Effect size	*P*-value
Age		55.27 ± 18.72	56.71 ± 19.73	57.15 ± 17.80	T test	0.136	0.870
Gender (n, %)	Male	39 (61.90)	26 (57.78)	22 (64.71)	Chi-squared	0.411	0.814
	Female	24 (38.10)	19 (42.22)	12 (35.29)			
Smoking history (n, %)	No	45 (71.43)	34 (75.56)	25 (73.53)	Chi-squared	0.230	0.891
	Yes	18 (28.57)	11 (24.44)	9 (26.47)			
History of Alcohol (n, %)	No	37 (58.73)	27 (60.00)	20 (58.82)	Chi-squared	0.020	0.990
	Yes	26 (41.27)	18 (40.00)	14 (41.18)			
Chronic lung disease (n, %)	No	58 (92.06)	42 (93.33)	31 (91.18)	Chi-squared	0.132	0.936
	Yes	5 (7.94)	3 (6.67)	3 (8.82)			
Diabetes (n, %)	No	68 (76.19)	29 (64.44)	24 (70.59)	Chi-squared	1.770	0.413
	Yes	15 (23.81)	16 (35.66)	10 (29.41)			
Chronic kidney disease (CKD stage ≥ 3)	No	51 (80.95)	36 (80.00)	27 (79.41)	Chi-squared	0.036	0.982
	Yes	12 (19.05)	9 (20.00)	7 (20.59)			
Malignant tumor (active stage) (n, %)	No	49 (77.78)	33 (73.33)	27 (79.41)	Chi-squared	0.467	0.792
	Yes	14 (22.22)	12 (26.67)	7 (20.59)			
hypertension (n, %)	No	42 (66.67)	29 (64.44)	23 (67.65)	Chi-squared	0.100	0.951
	Yes	21 (33.33)	16 (35.56)	11 (32.35)			
Type of pneumonia (n, %)	Hospital-acquired pneumonia	41 (65.08)	20 (44.44)	24 (70.59)	Chi-squared	6.793	0.033
	Ventilator-associated pneumonia	22 (34.92)	25 (55.56)	10 (29.41)			
Bacteremia	No	52 (82.54)	28 (62.22)	28 (82.35)	Chi-squared	6.3924	0.031
	Yes	11 (17.46)	17 (37.78)	6 (17.65)			
Pre-infection hospitalization days		12.10 ± 3.56	16.47 ± 3.59	12.54 ± 4.68	T test	54.633	<.001
Be admitted to the ICU (n, %)	No	11 (17.46)	2 (4.44)	8 (23.53)	Chi-squared	6.239	0.044
	Yes	52 (82.54)	43 (95.56)	26 (76.47)			
Mechanical ventilation (at the time of diagnosis) (n, %)	No	15 (23.81)	2 (4.44)	8 (23.53)	Chi-squared	7.867	0.020
	Yes	48 (76.19)	43 (95.56)	26 (76.47)			
APACHE II score		19.38 ± 5.62	25.24 ± 6.14	19.96 ± 7.27	T test	12.830	<.001
SOFA score		8.05 ± 1.04	10.17 ± 1.12	8.32 ± 0.91	T test	59.600	<.001

ICU, Intensive Care Unit; APACHE, Acute Physiology and Chronic Health Evaluation; SOFA, Sequential Organ Failure Assessment.

### 3.2 Analysis of the drug resistance profiles of the bacterial strains in the three groups of patients

After the analysis of the drug resistance spectrum of the strains through the VITEK 2 system, it was found that, as shown in **Table 2**, **Table 3**. Three groups were absolutely resistant to carbapenems: the resistance rate to imipenem/meropenem was over 97%, confirming that all were carbapenem-resistant Acinetobacter baumannii (CRAB). They were also widely resistant to β-lactam drugs: the resistance rate to ceftazidime/cefepime was over 97%, and to piperacillin/tazobactam was over 94%, indicating that third- and fourth-generation cephalosporins and penicillin derivatives were ineffective. They were completely resistant to quinolones: the resistance rate to ciprofloxacin/levofloxacin was over 88%, making them clinically useless. The sensitivity to sulbactam preparations determined the advantage of Group A: the resistance rate to cefoperazone/sulbactam was the lowest in Group A (30.16% vs 53.33% in Group B, *P* = 0.037), with a sensitivity rate as high as 71.43%. In the double-sensitivity (colistin + sulbactam) test, the proportion in Group A was 63.49%, significantly higher than that in Group B (46.67%), providing an ideal basis for combined treatment. Group B showed a higher resistance rate to tigecycline, 31.11%, significantly higher than that in Group A (26.98%) and Group C (2.97%). The core treatment regimen (polymyxin + tigecycline) was not very effective. The proportion of triple resistance (carbapenem + quinolone + aminoglycoside) was as high as 75.56%, reflecting a more severe multi-drug resistance background. Group C did not take advantage of the high sensitivity to tigecycline: the sensitivity rate to tigecycline was the highest (73.53%). The high sensitivity rate to cefoperazone/sulbactam (71.43%) in Group A was the microbiological basis for its therapeutic advantage, while the high resistance rate to tigecycline (31.11%) and the high proportion of triple resistance (75.56%) in Group B limited the effectiveness of the treatment plan.

**Table 2:** *In vitro* drug resistance results of three groups of strains (n, %).

Antibiotic types		Group A (n = 63)	Group B (n = 45)	Group C (n = 35)	Test	Effect size	*P*-value
Levofloxacin	Yes	57 (90.48)	42 (93.33)	30 (88.24)	Chi-squared	0.624	0.732
	No	6 (9.52)	3 (6.67)	4 (11.76)			
Ceftazidime	Yes	63 (100.00)	45 (100.00)	35 (100.00)	Chi-squared	–	–
	No	0 (0)	0 (0)	0 (0)			
Imipenem	Yes	62 (98.41)	44 (97.78)	33 (97.06)	Chi-squared	0.200	0.905
	No	1 (1.59)	1 (2.22)	1 (2.94)			
Piperacillin/tazobactam	Yes	62 (98.41)	43 (95.56)	32 (94.12)	Chi-squared	1.364	0.505
	No	1 (1.59)	2 (4.44)	2 (5.88)			
Ciprofloxacin	Yes	61 (96.83)	42 (93.33)	32 (94.12)	Chi-squared	0.770	0.681
	No	2 (3.17)	3 (6.67)	2 (5.88)			
Minocycline	Yes	20 (31.75)	18 (40.00)	11 (32.35)	Chi-squared	0.883	0.643
	No	43 (68.25)	27 (60.00)	23 (67.65)			
Tobramycin	Yes	48 (76.19)	37 (82.22)	26 (76.47)	Chi-squared	0.635	0.728
	No	15 (23.81)	8 (17.78)	8 (23.53)			
Meropenem	Yes	62 (98.41)	44 (97.78)	33 (97.06)	Chi-squared	0.200	0.905
	No	1 (1.59)	1 (2.22)	1(2.94)			
Cefepime	Yes	63 (100.00)	44 (97.78)	33 (97.06)	Chi-squared	1.690	0.430
	No	0 (0.00)	1 (2.22)	1 (2.94)			
Cefoperazone/sulbactam	Yes	19 (30.16)	24 (53.33)	11 (32.35)	Chi-squared	6.593	0.037
	No	44 (69.84)	21 (46.67)	23 (67.65)			
Doxycycline	Yes	24 (38.10)	20 (44.44)	13 (38.24)	T test	0.508	0.776
	No	39 (61.90)	25 (55.56)	21 (61.76)			
Compound trimethoprim/sulfamethoxazole	Yes	59 (93.65)	41 (91.11)	30 (88.24)	Chi-squared	0.853	0.653
	No	4 (6.35)	4 (8.89)	4 (11.76)			
Amikacin	Yes	40 (63.49)	33 (73.33)	22 (64.71)	Chi-squared	1.245	0.536
	No	23 (36.51)	12 (26.67)	12 (35.24)			
Tigecycline	Yes	17 (26.98)	14 (31.11)	1 (2.94)	T test	10.088	0.006
	No	46 (73.02)	31 (68.89)	33 (97.06)			
Colistin	Yes	3 (4.76)	5 (11.11)	4 (11.76)	T test	2.002	0.367
	No	60 (95.24)	40 (88.89)	30 (88.24)			

**Table 3:** The *in vitro* drug sensitivity of the main drugs to the three groups of patients (n, %).

Antibiotic types		Group A (n = 63)	Group B (n = 45)	Group C (n = 35)	Test	Effect size	*P*-value
Sensitive to any sulbactam formulation	No	18 (28.57)	22 (48.89)	17 (50.00)	Chi-squared	6.318	0.042
	Yes	45 (71.43)	23 (51.11)	17 (50.00)			
Tigecycline sensitive	No	35 (55.56)	27 (60.00)	9 (26.47)	Chi-squared	10.107	0.006
	Yes	28 (44.44)	18 (40.00)	25 (73.53)			
Susceptible to colistin	No	3 (4.76)	5 (11.11)	5 (14.71)	Chi-squared	2.929	0.231
	Yes	60 (95.24)	40 (88.89)	29 (85.24)			
Dual sensitivity (sulfamethoxazole + sulbactam)	No	23 (36.51)	24 (53.33)	18 (52.94)	Chi-squared	3.919	0.141
	Yes	40 (63.49)	21 (46.67)	16 (47.06)			
Triple drug resistance (carbapenem + quinolone + aminoglycoside)	No	29 (46.03)	11 (24.44)	16 (47.06)	Chi-squared	6.209	0.045
	Yes	34 (53.97)	34 (75.56)	18 (52.94)			

### 3.3 28-day all-cause mortality rate

The crude 28-day all-cause mortality rates were 39.68% (25/63) in Group A, 60.00% (27/45) in Group B, and 64.71% (22/34) in Group C, with a significant difference among the three groups (*p* = 0.028, **Table 4**).

**Table 4:** 28-day all-cause mortality rate.

Group	Deaths/Total	Mortality rate (%)	*p* value
A	25/63	39.68	Reference
B	27/45	60.00	0.037 vs A
C	22/34	64.71	0.019 vs A

The P-values were obtained from the chi-square test. The comparison between B and A had a P-value of 0.037, and the comparison between C and A had a P-value of 0.019.

### 3.4 Evaluation of serum inflammatory markers and immune function in three groups of patients

The baseline PCT and CRP levels in Group B (polymyxin + tigecycline) were significantly higher than those in Group A (PCT: 8.27 vs 4.68 μg/L, *P* < 0.001; CRP: 172.25 vs 135.15 mg/L, *P* < 0.001), which were consistent with their higher APACHE II/SOFA scores, VAP rates, and bacteremia rates. On the 7th day of treatment, the PCT in Group A dropped to 1.19 μg/L, significantly lower than that in Group B (2.41 μg/L) and Group C (3.76 μg/L) (*P* < 0.001). The CRP level dropped to 45.85 mg/L on the 7th day, significantly lower than that in Group C (73.22 mg/L) (*P* < 0.001), indicating the best anti-inflammatory effect and significant differences in treatment response. In Group C (other treatment regimens), the inflammatory control was poor: the reduction in PCT and CRP on the 7th day was the smallest, and it was significantly higher than that in Group A, suggesting insufficient effectiveness of the treatment regimens. The reduction in Group B (polymyxin + tigecycline) was significant, with the baseline level being the highest (8.27 μg/L) but the PCT dropping from 8.27 to 2.41 μg/L (a 70% reduction), reflecting its anti-inflammatory effect on critically ill patients. Through immune function assessment, it was found that Group A had the best immune status: the CD4^+^/CD8^+^ ratio was the highest (1.21). The NK cell level (0.10 × 10^9^/L) was significantly higher than that in Group B (0.07 × 10^9^/L, *P* <.001), indicating that Group A had a stronger innate immune surveillance function. While Group C had the most significant cellular immunosuppression, with the CD4^+^/CD8^+^ ratio being the lowest (0.9), which corresponded to its high mortality rate (64.71%). Group A had significantly lower NK cells compared to Group C and the other groups (0.07 vs 0.10/0.08 × 10^9^/L, *P* < 0.001). The regimen containing sulbactam (Group A) had the best anti-inflammatory efficacy: significantly reducing PCT/CRP levels and having the least immunosuppression. Polymyxin + tigecycline (Group B) was effective for critically ill patients: although the baseline inflammatory level was the highest, it significantly decreased on the 7th day, suggesting that this regimen is still applicable to high-risk populations. Other regimens (Group C) were less effective: poor inflammatory control and severe immunosuppression, consistent with the highest mortality rate (**Table 5**).

**Table 5:** Evaluation of serum inflammatory markers and immune function in three groups of patients ( $\bar{x}\pm \text{ s}$).

Parameters		Group A (n = 63)	Group B (n = 45)	Group C (n = 35)	Test	Effect size	*P*-value
PCT (μg/L)	baseline	4.68 ± 0.54	8.27 ± 1.14*	4.82 ± 1.09^	T test	237.300	<.001
	3 days	2.95 ± 0.92	5.75 ± 0.87*	3.47 ± 0.68^#^^	T test	148.173	<.001
	7 days	1.19 ± 0.28	2.41 ± 0.35*	3.76 ± 0.53^#^^	T test	532.757	<.001
CRP (mg/L)	baseline	135.15 ± 20.05	172.25 ± 25.82*	141.95 ± 15.08^	T test	42.852	<.001
	3 days	86.43 ± 9.54	118.55 ± 12.42*	97.14 ± 17.48^#^^	T test	119.863	<.001
	7 days	45.85 ± 5.83	62.55 ± 6.07*	73.22 ± 6.33^#^^	T test	248.973	<.001
CD3+ (×10^9^/L)		0.41 ± 0.12	0.45 ± 0.11^	0.38 ± 0.09	T test	4.476	0.013
CD3+CD4+ (×10^9^/L)		0.28 ± 0.52	0.25 ± 0.14	0.22 ± 0.08^#^	T test	4.921	0.009
CD3+CD8+ (×10^9^/L)		0.20 ± 0.09	0.18 ± 0.06	0.18 ± 0.07	T test	0.950	0.389
CD19+ (×10^9^/L)		0.07 ± 0.02	0.05 ± 0.02*	0.06 ± 0.03^#^	T test	11.706	<.001
CD3^-^CD16^+^CD56^+^ (×10^9^/L)		0.10 ± 0.03	0.07 ± 0.03*	0.08 ± 0.04^	T test	11.926	<.001
CD4^+^/CD8^+^ (×10^9^/L)		1.21 ± 0.09	1.00 ± 0.09*	0.90 ± 0.08^#^^	T test	150.395	<.001

PCT is procalcitonin, CRP is C-reactive protein. ^*^ Indicates a difference between Group B and Group A. ^#^ Indicates a difference between Group C and Group A. ^^^ Indicates a difference when compared with Group B. *P* < 0.05.

### 3.5 Propensity score matching analysis

To further eliminate baseline differences, we performed 1:1 nearest-neighbor propensity score matching (caliper = 0.2) between Group A and Group B, and separately between Group A and Group C. Matching variables included age, APACHE II score, SOFA score, VAP status, ICU admission, mechanical ventilation, and bacteremia. After matching, baseline characteristics were well balanced (all standardized differences<0.1, see **Supplementary Table 1**).

As presented in **Table 6**, in the matched A–B cohort (42 pairs), the 28-day mortality was 40.5% (17/42) in Group A vs 57.1% (24/42) in Group B (*P* = 0.042). In the matched A–C cohort (35 pairs), mortality was 37.1% (13/35) in Group A vs 62.9% (22/35) in Group C (*P* = 0.008). These results confirm that sulbactam-based regimens provide a survival benefit independent of baseline severity.

**Table 6:** Comparison of 28-day mortality rates after propensity score matching.

Matched cohort	Number of pairs	Group A mortality	Group B/C mortality	*P* value
A vs B	42	40.5% (17/42)	57.1% (24/42)	0.042
A vs C	35	37.1% (13/35)	62.9% (22/35)	0.008

The P-values are derived from the McNemar test (paired chi-square) or conditional Logistic regression.

### 3.6 Subgroup analysis

We performed subgroup analyses to assess the consistency of treatment effects across different risk strata. As shown in **Table 7**: In patients with APACHE II ≥25 (n=61):Mortality was 77.8% (14/18) in Group A, 88.9% (24/27) in Group B, and 87.5% (14/16) in Group C. The difference did not reach statistical significance (*P* = 0.312), likely due to reduced sample size. In VAP patients only (n=57):Mortality was 45.5% (10/22) in Group A, 68.0% (17/25) in Group B, and 70.0% (7/10) in Group C (*P* = 0.048). The survival benefit of Group A remained significant in this high-risk subgroup.

**Table 7:** Results of subgroup analysis.

Subgroup	Group A mortality	Group B mortality	Group C mortality	*P* value
APACHE II ≥25 (n=61)	77.8% (14/18)	88.9% (24/27)	87.5% (14/16)	0.312
VAP only (n=57)	45.5% (10/22)	68.0% (17/25)	70.0% (7/10)	0.048

The P value is derived from the chi-square test (stratified analysis).

### 3.7 Logistic analysis of risk factors for mortality

Using binary logistic regression (dependent variable: 28-day all-cause mortality), we first performed univariate analysis to select candidate variables. Independent predictive value was then focused on verifying APACHE II score, SOFA score, and CD4^+^/CD8^+^ ratio (all as continuous variables). After adjusting for these potential confounders, treatment group remained an independent predictor of mortality (**Tables 8**, **9**). Compared with Group A, Group B had an 82% higher risk of death (adjusted OR = 1.82, 95% CI: 1.07–3.11,*P* = 0.028), and Group C had a 115% higher risk (adjusted OR = 2.15, 95% CI: 1.21–3.83,*P* = 0.009). The CD4^+^/CD8^+^ ratio was also an independent predictor; after re-scaling to a 0.1-unit increase, the adjusted OR was 0.52 (95% CI: 0.36–0.75,*P* = 0.004), indicating that a higher ratio (i.e., better immune function) was associated with a significantly lower risk of death.

**Table 8:** Logistic analysis of risk factors for mortality.

Variable	Adjusted OR	95% CI	*P* value
Treatment group			
Group B (vs A)	1.82	1.07 – 3.11	0.028
Group C (vs A)	2.15	1.21 – 3.83	0.009
APACHE II (per 1 point)	2.02	1.49 – 2.76	<0.001
SOFA (per 1 point)	8.99	2.44 – 33.19	0.001
CD4^+^/CD8^+^ (per 0.1)	0.52	0.36–0.75	0.004

**Table 9:** Univariate regression analysis.

Parameters	Wald	df	Sig.	Exp(B)	95% CI	
					Lower	upper
Type of pneumonia (n, %)	0.668	1	0.414	0.776	0.013	6.015
Bacteremia consolidation	0.801	1	0.371	5.566	0.130	238.675
Pre-infection hospitalization days	3.513	1	0.061	0.027	0.001	1.179
Be admitted to the ICU (n, %)	0.249	1	0.618	0.788	0.309	2.007
Mechanical ventilation (at the time of diagnosis) (n, %)	0.262	1	0.608	3.139	0.039	249.754
APACHEII/10	7.821	1	0.005	3.574	1.464	8.727
SOFA/10	4.087	1	0.043	24.962	1.103	564.861
PCT (μg/L) baseline	0.172	1	0.679	0.772	0.227	2.624
PCT (μg/L) 3 days	2.939	1	0.086	0.223	0.040	1.2401
PCT (μg/L) 7 days	1.109	1	0.292	4.431	0.277	70.796
CRP (mg/L) baseline	0.065	1	0.798	0.469	0.001	155.172
CRP (mg/L) 3 days	3.563	1	0.059	0.000	0.000	1.912
CRP (mg/L) 7 days	1.037	1	0.309	0.265	0.020	3.420
CD3+ (×10^9^/L)	0.010	1	0.918	1.696	0.000	41644.124
CD3+CD4+ (×10^9^/L)	2.548	1	0.110	0.054	0.002	1.943
CD19+ (×10^9^/L)	0.288	1	0.592	31.101	0.000	8838000.333
CD3^-^CD16^+^CD56^+^ (×10^9^/L)	1.573	1	0.210	0.003	0.000	25.454
CD4+/CD8+	4.073	1	0.044	0.482	0.28–0.82	0.044
Tigecycline	0.048	1	0.826	1.499	0.041	55.096

## 4 Discussion

In the fields of critical care medicine and infectious diseases, multidrug-resistant Acinetobacter baumannii (MDRAB) pneumonia remains a highly challenging clinical problem. This Gram-negative bacterium exhibits a complex resistance mechanism, including the production of OXA-type carbapenemases (such as OXA-23/40/58), overexpression of efflux pumps (AdeABC system), absence of membrane channel proteins, and modification of lipopolysaccharides, resulting in a resistance rate to carbapenems exceeding 70%. In some regions, pan-resistant strains (XDRAB) have even emerged, making clinical treatment options extremely limited (Chen et al., 2018; Castanheira et al., 2023; Nguyen and Joshi, 2025). Epidemiological studies have shown that the in-hospital mortality rate of patients with multi-drug resistant Acinetobacter baumannii (MDR-AB) pneumonia (15.46%) is higher than that of patients without multi-drug resistant infections (5.88%). The average length of hospital stay for MDR-AB patients is also longer (13.4 days versus 10.5 days), and the average hospitalization cost is also higher (32,086 US dollars versus 24,367 US dollars) (Lapensee et al., 2014). Based on retrospective research evidence, this article integrates treatment strategies and risk factors for death to provide a basis for clinical decision-making. The current treatment strategy mainly involves combined medication, and treatment regimens based on sulbactam compound preparations (such as cefoperazone/sulbactam) have significant survival advantages (Niu et al., 2019; Kanchanasuwan et al., 2021). The mechanism lies in the dual effects of sulbactam: Firstly, it irreversibly inhibits β-lactamases, restoring the antibacterial activity of β-lactam drugs; Secondly, it directly binds to penicillin-binding proteins (PBPs), interfering with the synthesis of the cell wall (Akova, 2008; Arer and Kar, 2023; Boattini et al., 2025). A 2024 meta-analysis of 10 retrospective studies concluded that the treatment regimen based on cefoperazone/sulbactam was superior to other non-cefpoperazone/sulbactam treatment regimens in the case of multidrug-resistant Acinetobacter baumannii infections, demonstrating higher survival rates and clinical improvement effects (Huang et al., 2024). The combination regimens of polymyxins should be selected on an individual basis based on the results of drug sensitivity tests. The combination therapy of colistin and carbapenems is superior to that of colistin and tigecycline in the treatment of pan-drug-resistant Acinetobacter baumannii (XDR-AB) infections. The 14-day mortality rates were 15% and 35%, respectively (Cheng et al., 2015). It is worth noting that in a *post hoc* analysis, patients who were treated with tigecycline and whose isolated bacteria had a minimum inhibitory concentration (MIC) greater than 2 milligrams per liter had a significantly higher mortality rate than those with an MIC equal to or less than 2 milligrams per liter. The mortality rate of this subgroup was much higher (10 out of 12 died, while 37 out of 84 died; *P* = 0.01) (Chuang et al., 2014). Minocycline, as a representative of “old drugs with new uses”, also shows potential in combination therapy. A study involving 55 patients with MDR-AB infections reported that the clinical success rate of minocycline monotherapy or combination therapy was 73%, the bacterial clearance rate was 78%, but the infection-related mortality rate still reached 25% (Goff et al., 2014). Its advantage lies in its stable antibacterial activity against non-fermenting Gram-negative bacilli (including Acinetobacter baumannii), with a drug resistance rate lower than 20%, making it particularly suitable for infections caused by OXA-23 type strains (Uskudar-Guclu et al., 2024).

The World Health Organization (WHO) has classified it as a “key priority” pathogen. The mortality rate of hospital-acquired pneumonia (HAP) and ventilator-associated pneumonia (VAP) caused by it can be as high as 30% to 70%, posing a particularly lethal threat to critically ill patients with multiple organ failure (Inchai et al., 2015). Over the years, the treatment strategies have focused on optimizing combination therapies (such as sulbactam-based preparations combined with polymyxins or tigecycline) and the development of new drugs (such as sulbactam-dulobactam, eravacycline) (Karakonstantis et al., 2021). The sulbactam-Dulobatam combination approved by the FDA in 2023 significantly reduced the MIC value of sulbactam by inhibiting type D β-lactamases. The ATTACK clinical trial demonstrated that it could reduce the 28-day mortality rate of pneumonia patients by 13.2% (Kaye et al., 2023).

In this context, this study retrospectively analyzed 142 patients with MDRAB pneumonia who were admitted to our hospital from February 2022 to February 2025. The aim was to evaluate the clinical benefits of different treatment strategies and identify independent risk factors for mortality. The patients were divided into three groups: Group A (n=63) received a combined regimen based on sulbactam complex preparation (cefoperazone/sulbactam) (combined with polymyxin or tigecycline); Group B (n=45) used a polymyxin class + tigecycline combined regimen; Group C (n=34) was given other regimens (including single drugs or non-standard combinations). The core findings showed that the 28-day mortality rate of the three groups increased in a stepwise manner: Group A (39.7%) was significantly lower than Group B (60.0%) and Group C (64.7%), with a mortality risk of 60.00% for Group B and an increase of 64.71% for Group C compared to Group A (*P* = 0.028). The underlying mechanism of this difference can be analyzed from three aspects: The synergistic effect of pharmacokinetics/pharmacodynamics (PK/PD) advantages is the core of the excellent efficacy of Group A. The concentration of cefoperazone/sulbactam in lung tissue can reach 40-60% of the blood drug concentration. Sulbactam can target the penicillin-binding protein PBP2 of Acinetobacter baumannii. When combined with polymyxin, the latter significantly enhances the intracellular penetration of sulbactam by destroying the bacterial outer membrane lipopolysaccharide layer (Zhu et al., 2023; Nicolau et al., 2025). In this study, Group A directly demonstrated the best inflammation control: on the 7th day of treatment, PCT in Group A (sulbactam regimen) dropped to 1.19 μg/L, significantly lower than that in Group B (2.41 μg/L) and Group C (3.76 μg/L) (P< 0.001). CRP dropped to 45.85 mg/L on the 7th day, significantly lower than that in Group C (73.22 mg/L) (P< 0.001). This result is highly consistent with a study on the treatment of polymyxin E published by Amani Yehya et al. in 2025, which confirmed that aerosol combined with intravenous administration can increase the local drug concentration in the lungs (Yehya et al., 2025). Robustness of the findings despite baseline imbalances. A legitimate concern is that Group B had significantly higher APACHE II and SOFA scores, more VAP, and higher ICU admission rates at baseline, which could bias the unadjusted mortality comparison. To address this, we employed two complementary approaches: (1) propensity score matching that balanced all measured confounders, and (2) multivariable logistic regression that included treatment group along with disease severity indices. Both methods consistently demonstrated that sulbactam-based combination therapy (Group A) was associated with significantly lower 28-day mortality compared with Group B and Group C. Therefore, the observed survival advantage of Group A is unlikely to be explained solely by its less severe baseline condition. Nevertheless, we acknowledge that unmeasured confounders (e.g., timing of appropriate therapy, source control, or prior antibiotic exposure) could still exist.

The discrepancy in the match between the drug resistance spectrum and the treatment strategy further explains the gap in therapeutic efficacy between the groups. The treatment plan for group B has two issues. Firstly, the resistance rate of tigecycline to the bacteria in this group is as high as 31.11% (compared to only 26.98% in group A); secondly, the penetration rate of polymyxins in lung tissue is only 20-30%, while 95.56% of patients in group B require mechanical ventilation (as pulmonary edema further reduces drug penetration). When the bacteria are resistant to any drug, the mortality rate of group B surges to 60.00% (27/45), while group A maintains a survival rate of 60.32% (38/63) due to the synergistic buffering effect of sulbactam, even though the polymyxins are resistant (compared to 40.00% in group A). The mortality rate of group C is as high as 64.71%, and it did not utilize the advantage of the tigecycline sensitivity rate of its strain (73.53% vs 40.00% in group A). This is in sharp contrast to a study by Youfa Qin et al. in 2018: Tigecycline combined with high-dose cefoperazone-sulbactam significantly improved the overall treatment success rate of ventilator-associated pneumonia caused by extensively drug-resistant Acinetobacter baumannii (85.7% vs 47.6%) (Qin et al., 2018). The practical deviations in Group C highlight the clinical necessity of antimicrobial susceptibility-guided therapy (AST), especially in cases of pan-resistant infections.

This study is the first to systematically evaluate the baseline immune status of patients with MDR-AB pneumonia. Flow cytometry analysis revealed that group A exhibited the best ability to control inflammation. On the 7th day of treatment, PCT in group A dropped to 1.19 μg/L, significantly lower than that in group B (2.41 μg/L) and group C (3.76 μg/L) (*P* < 0.001). This is consistent with the results of previous studies, as the sulbactam-containing regimen can reduce pro-inflammatory factor levels more rapidly (Spernovasilis et al., 2025). It is worth noting that although the baseline PCT level was the highest in Group B (8.1 μg/L), it decreased by 70%, suggesting that polymyxin + tigecycline still has anti-inflammatory effects on critically ill patients, but the effect is not as good as that of Group A. Group A had the best preservation of immune function, with an average CD4^+^/CD8^+^ ratio of 1.21, significantly higher than 0.9 in Group C (*P* < 0.001). The number of NK cells (0.10 × 10^9^/L) was also higher than that in Group B (0.08 × 10^9^/L, *P* < 0.001). In our multivariable analysis, the CD4^+^/CD8^+^ ratio was a strong protective factor. Because the ratio typically ranges from 0.2 to 2.5, we re-scaled the variable to a 0.1-unit increment for clinical interpretability. Each 0.1-unit increase in the CD4^+^/CD8^+^ ratio was associated with a 48% reduction in mortality risk (adjusted OR = 0.52, 95% CI: 0.36–0.75, *P* = 0.004). This finding aligns with Li et al., who reported that a low CD4^+^/CD8^+^ ratio indicates T-cell exhaustion and impaired protective immunity (Vassallo et al., 2025).

This study constructed a “dual-pillar mortality risk model” for MDRAB pneumonia through multi-factor analysis: the disease severity pillar (invariable factors): APACHE II (adjusted HR = 2.042) and SOFA (HR = 8.993) are the strongest predictors of mortality. These results are highly consistent with the current treatment trends. A review published in 2025 emphasized that the treatment of hospital-acquired pneumonia caused by CRAB with sulbactam-dolobactam has a good lung tissue penetration rate (sulbactam about 50%, dolobactam about 38%), and its efficacy is superior to polymyxin E, supporting it as a first-line treatment option. The pharmacokinetics of this drug in the lungs indicate its good distribution (high permeability of the intercellular fluid of epithelial cells), and it has fewer side effects, while meropenem prolonging the infusion can enhance the time-dependent bactericidal effect (Franzone et al., 2024). For extremely high-risk patients (with APACHE II score ≥ 25 or SOFA score ≥ 9), a three-drug combination therapy can be considered: for instance, the new generation of polymyxin MRX-8 (with lower nephrotoxicity than traditional polymyxins) combined with meropenem, to achieve a “double insurance” strategy by inhibiting the formation of drug-resistant subgroups (Heil et al., 2023).

### 4.1 Limitations

However, this study has three limitations: the retrospective design cannot fully control for confounding bias. Although PSM and multivariable adjustment were employed, the significantly higher baseline disease severity in Group B (APACHE II 25.24 vs. 19.38 in Group A) means that a direct crude mortality comparison would overestimate the relative benefit of Group A. By using PSM and multivariate models, we attempted to minimize this bias; however, residual confounding from unmeasured factors (e.g., timing of appropriate antibiotics, adequacy of source control, or prior antibiotic exposure) cannot be entirely excluded. Secondly, the detection of drug resistance genotypes (such as blaOXA-23/blaNDM-1) was not conducted, making it difficult to analyze the association between molecular mechanisms and therapeutic efficacy. Thirdly, the sample size calculation was originally based on a two-arm comparison. The actual three-group, unbalanced design reduces statistical power for certain pairwise comparisons, especially for the smaller Group C (n = 34). Nevertheless, the consistency of results across multiple analytical approaches (multivariable regression, propensity score matching, and subgroup analyses) supports the robustness of our main conclusions. Finally, new drugs such as sulbactam-dulobactam have not been included in the protocol, and their benefits in the local population need to be verified by future prospective studies. Due to the retrospective and non-randomized design, causality cannot be inferred. The observed associations should be considered hypothesis-generating and require validation in prospective studies.

## Data Availability

The data supporting the findings of this study can be obtained from the corresponding author upon request.
