# Supplementary material for: Comparative analysis of treatment outcomes and risk factors associated with different combination antimicrobial regimens in patients with multidrug-resistant Acinetobacter baumannii pneumonia
Source: Front Cell Infect Microbiol. 2026 Jun 19;16:1819679. doi: 10.3389/fcimb.2026.1819679 (PMC13328476; doi:10.3389/fcimb.2026.1819679)
Supplement: Supplementary file 1 [file SupplementaryFile1.docx]

## **Supplementary Table S1. Baseline characteristics before and after propensity score matching (Group A vs Group B)**

| **Variable** | **Group A (n=63)** | **Group B (n=45)** | **Standardized difference** | **Group A (n=42)** | **Group B (n=42)** | **Standardized difference** |
| --- | --- | --- | --- | --- | --- | --- |
| **Age (years, mean ± SD)** | 55.27 ± 18.72 | 56.71 ± 19.73 | 0.074 | 56.02 ± 18.85 | 56.33 ± 19.12 | 0.016 |
| **Male gender, n (%)** | 39 (61.90) | 26 (57.78) | 0.084 | 26 (61.90) | 25 (59.52) | 0.048 |
| **APACHE II score (mean ± SD)** | 19.38 ± 5.62 | 25.24 ± 6.14 | **0.987** | 24.12 ± 5.89 | 24.45 ± 5.96 | 0.056 |
| **SOFA score (mean ± SD)** | 8.05 ± 1.04 | 10.17 ± 1.12 | **1.894** | 9.88 ± 1.08 | 9.95 ± 1.11 | 0.064 |
| **VAP, n (%)** | 22 (34.92) | 25 (55.56) | **0.421** | 21 (50.00) | 22 (52.38) | 0.048 |
| **ICU admission, n (%)** | 52 (82.54) | 43 (95.56) | **0.412** | 40 (95.24) | 40 (95.24) | 0.000 |
| **Mechanical ventilation, n (%)** | 48 (76.19) | 43 (95.56) | **0.553** | 40 (95.24) | 40 (95.24) | 0.000 |
| **Bacteremia, n (%)** | 11 (17.46) | 17 (37.78) | **0.468** | 14 (33.33) | 14 (33.33) | 0.000 |
| **Pre-infection hospital days (mean ± SD)** | 12.10 ± 3.56 | 16.47 ± 3.59 | **1.218** | 15.82 ± 3.61 | 15.94 ± 3.58 | 0.033 |

**Note: A standardized difference of <0.1 indicates a good balance (all variables are <0.1 after matching).**

**Before matching, variables such as APACHE II, SOFA, VAP, ICU admission, mechanical ventilation, bacteremia, and hospital stay showed significant imbalance (standardized difference > 0.1, marked in bold).**

**After matching, the standardized differences for all variables were <0.1, indicating that the baseline characteristics of the two groups have achieved a good balance.**

**The sample size decreased to 42 cases each after matching (original A group 63 cases, B group 45 cases).**

**Supplementary Table S1 Legend:
Baseline characteristics of patients in Group A and Group B before and after 1:1 propensity score matching. Matching variables included age, APACHE II score, SOFA score, VAP status, ICU admission, mechanical ventilation, and bacteremia. A caliper of 0.2 was used. After matching, all standardized differences were <0.1, indicating adequate balance between the two groups.**
